# Partial hepatectomy for primary hepatic melanoma: a report of two cases and review of the literature

**DOI:** 10.1186/1477-7819-12-362

**Published:** 2014-11-28

**Authors:** Yuhua Zhang, Zhiming Hu, Weiding Wu, Jie Liu, Defei Hong, Chengwu Zhang

**Affiliations:** The hepatobiliary, Pancreatic and Minimal Invasive Surgery Department, Zhejiang Provincial People’s Hospital, 158# Shangtang Road, Hangzhou, Zhejiang 310014 China

**Keywords:** Primary hepatic melanoma, Diagnosis, Surgery, Prognosis

## Abstract

Malignant melanoma is an extremely aggressive cancer arising from melanocytes, associated with the development of metastases in up to 20% of patients. Although the liver is a frequent metastatic site of malignant melanoma, primary hepatic melanoma (PHM) is rare. The treatment of PHM is controversial, and the prognosis for affected patients remains poor. We present two PHM patients who underwent partial hepatectomy at our institution and review the clinical and pathological data from these cases. Our results suggest that it is difficult to make a preoperative diagnosis of PHM without pathological results. For patients with resectable PHM, surgical resection is a potentially curative treatment.

## Background

The American Cancer Society estimated that in 2012, 76,250 new cases of melanoma were diagnosed in the United States and 9,180 patients died from the disease [1]. Ninety percent of all melanomas are of cutaneous origin [2]. Although the liver is a frequent site of metastases from melanomas, primary hepatic melanoma (PHM) is rare. In this report, we present two patients with PHM who underwent partial hepatectomy in our institution and review the literature addressing this topic.

## Case presentation

### Case 1

**Figure 1:**
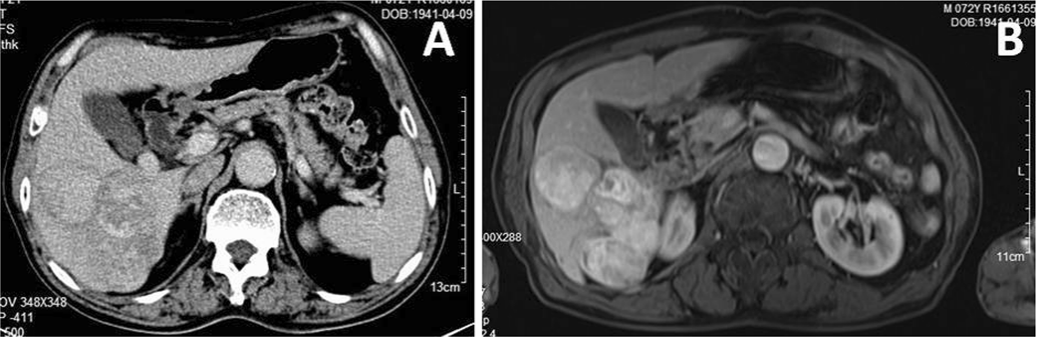
**Imaging findings of case 1. (A)**, **(B)** Computed tomography and magnetic resonance imaging showing a mass in the right liver lobe.

A 60-year-old male presented with a 1-month history of upper abdominal discomfort. Physical examination found no palpable lymph nodes, scars or pigmented patches. The abdomen was soft with no tenderness and no palpable liver or spleen. There was no history of an excised pigmented lesion or history of eye surgery. Liver function tests did not show signs of liver injury (alanine aminotransferase: 25 U/L, aspartate transaminase: 39 U/L, albumin: 36.4 g/L). The tumour markers alpha-fetoprotein (AFP) (3.49 μg/L), carcinoembryonic antigen (2.04 μg/L), carbohydrate antigen 19 to 9 (12.8 U/mL) as well as complete blood count (red blood cells: 4.82 × 10^12^/L, white blood cells: 7.37 × 10^9^/L, platelets: 223 × 10^9^/L) were all in the normal range. Abdominal ultrasonography revealed a mass in the right lobe of the liver with liquid at its centre. Computed tomography (CT) showed a 14 × 14 cm mixed-attenuation mass with a pseudocapsule and central low attenuation in the right lobe of the liver (Figure 1A). Magnetic resonance imaging (MRI) demonstrated an isointense high-signal lesion in the right lobe of the liver on T1-weighted imaging (T1W1), with mixed low- and high-signal intensity on T2-weighted imaging (T2W1) (Figure 1B). The mass showed peripheral enhancement with a contrast agent during the arterial and portal venous phases on both CT and MRI.

**Figure 2:**
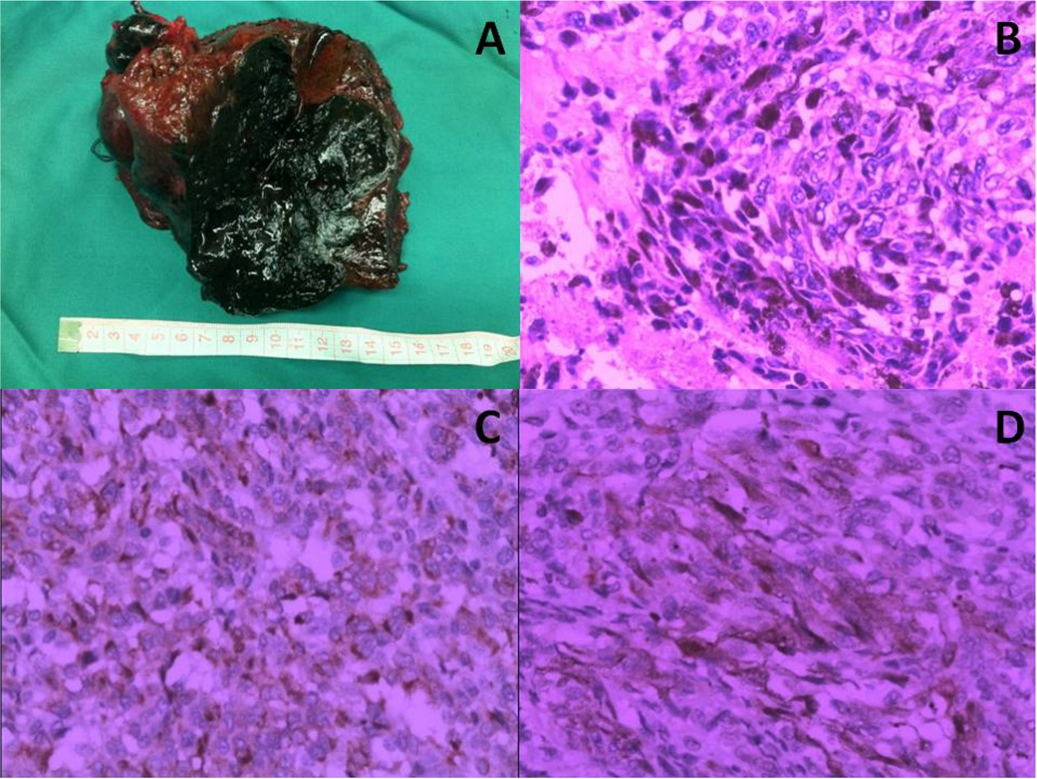
**Pathological findings of case 1. (A)** A black solid-cystic tumour was found in the right hemi-hepatectomy specimen. **(B)** The mass contained elongated malignant cells with + melanin pigment (×200). **(C)** The malignant cells were immunostained with HMB45 (×400). **(D)** The malignant cells were immunostained with S100 (×400).

The patient underwent right hemi-hepatectomy and resection of the lymph nodes in the hepatoduodenal ligament. A 15 × 15 cm solid-cystic tumour was found in the right lobe of the liver, and metastasized lymph nodes were found in the hepatoduodenal ligament (Figure 2A). The tumour had ruptured preoperatively, and intraperitoneal haemorrhage was found in the region of the right liver during the operation. Microscopic examination revealed the mass to contain elongated malignant cells arranged in a fascicular pattern and associated with melanin pigment (Figure 2B). Postoperative immunostaining of the liver tumour established the diagnosis of malignant melanoma; the tumour was focally strong-positive for HMB45 and diffuse-positive for S100 (Figure 2C,D), with no staining of cytokeratin (CK) and AFP. The patient had no history of melanocytic or pigmented lesions. No other melanomas were identified despite rigorous physical examination, which included anal and ophthalmologic examination in addition to a complete cutaneous evaluation. Further tests, including ophthalmoscopy, examination of the anogenital region, and upper and lower gastrointestinal endoscopy, were all normal. The patient recovered well and received interleukin-2 (IL-2) therapy postoperatively, but died 4 months after surgery from intra-peritoneal recurrence of the tumour.

### Case 2

**Figure 3:**
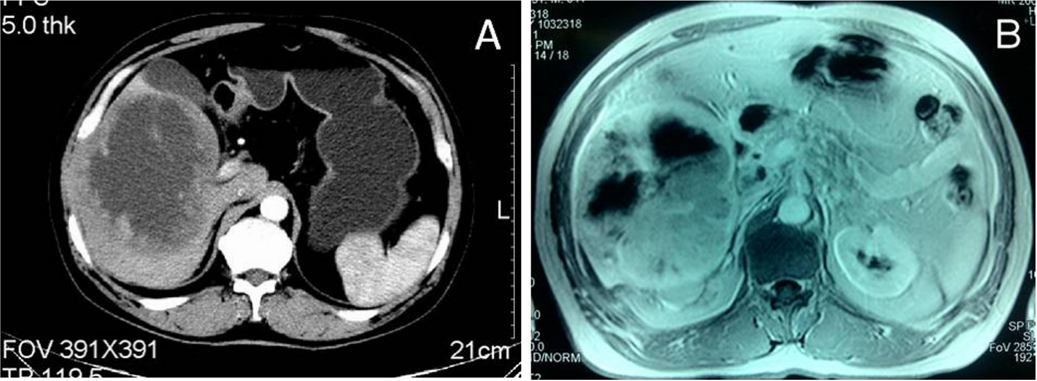
**Imaging findings of case 2. (A)**, **(B)** Computed tomography and magnetic resonance imaging showing multiple lesions in the right liver lobe.

A 72-year-old male patient was admitted to our hospital because of a right hepatic lesion found on ultrasound scan; he did not have abdominal discomfort, fever or weight loss. Tumour markers (AFP: 2.4 μg/L, carcinoembryonic antigen: 3.6 μg/L and carbohydrate antigen 19 to 9: 2.9 U/mL), complete blood count (red blood cells: 4.74 × 10^12^/L, white blood cells: 6.23 × 10^9^/L, platelets: 181 × 10^9^/L) and liver function tests (alanine aminotransferase: 11 U/L, aspartate transaminase: 25 U/L, albumin: 39.4 g/L) were all within normal limits. Computed tomography demonstrated multiple high-attenuation lesions in the right lobe of the liver (Figure 3A). MRI showed a heterogeneous high-signal mass on T2W1 with a central low-signal area in the right lobe of the liver; the centre of the lesion had low signal intensity on T1W1 (Figure 3B). With a contrast agent, the mass showed enhancement in the arterial phase and no enhancement in the portal venous phase.

**Figure 4:**
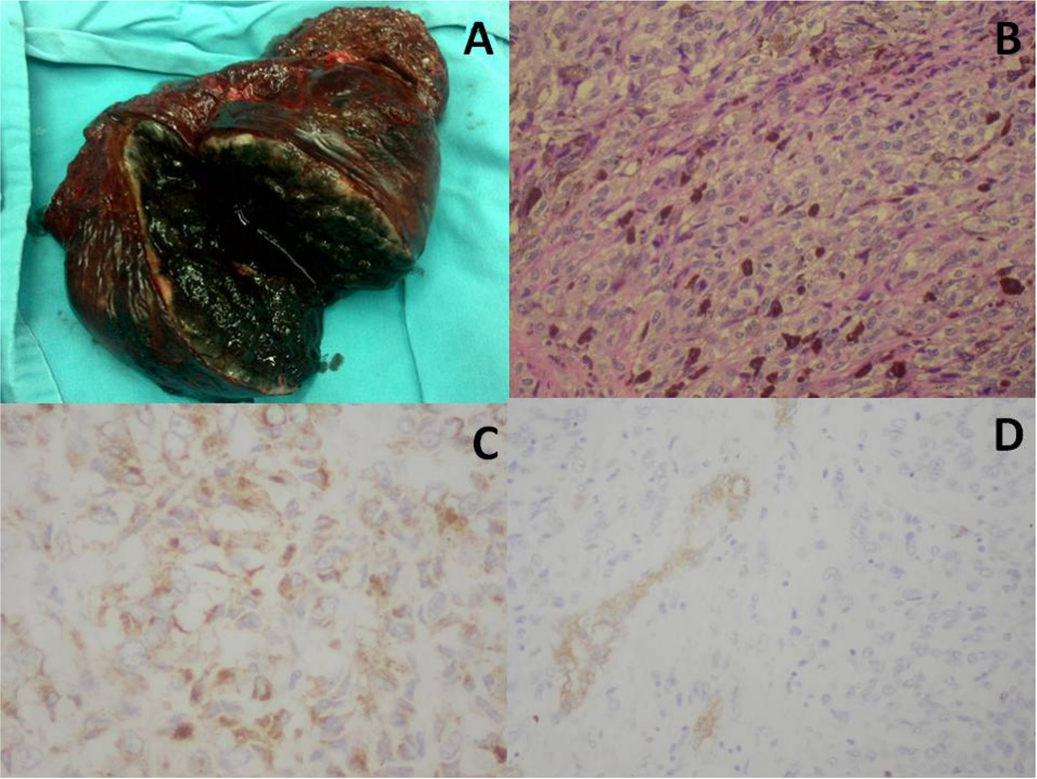
**Pathological findings of case 2. (A)** Multiple black solid-cystic lesions were found in the right hemi-hepatectomy specimen. **(B)** The mass contained elongated malignant cells with melanin pigment (×400). **(C)** The malignant cells were immunostained with HMB45 (×400). **(D)** The malignant cells were immunostained with S100 (×400).

The patient underwent right hemi-hepatectomy as well as lymph node resection in the hepatoduodenal ligament. Gross pathologic examination demonstrated a 14 × 12 cm solid-cystic lesion in the right liver (Figure 4A) and enlarged, black lymph nodes in the hepatoduodenal ligament. Microscopic examination revealed the lesion to be full of elongated malignant cells (Figure 4B), and the lesion was positive on immunostaining with HMB45 and S100 (Figure 4C,D), negative with CK and AFP. Based on these results, the patient was diagnosed with malignant melanoma. No other melanoma sites were detected despite rigorous physical examination, including anal and ophthalmologic examination in addition to complete cutaneous evaluation. Further tests, including positron emission tomography-CT (PET-CT), ophthalmoscopy, examination of the anogenital region, and upper and lower gastrointestinal endoscopy, were all normal. An uneventful postoperative recovery was achieved, and the patient was treated with IL-2. The patient has experienced a 12-month disease-free interval up to the present.

## Discussion

Malignant melanoma is an extremely aggressive neoplasm of melanocytes [3]. It usually originates in the epithelial tissues of the skin, retina and anorectal canal, but it sometimes originates in rare primary locations such as the gastrointestinal tract, genital tract, accessory nasal cavity and parotid. Primary hepatic melanoma is an extremely rare neoplasm; to our knowledge, only 12 cases have been reported. The origin of PHM is unclear, as there are no melanocytes in the liver. Some authors have suggested that these neoplasms arise from ectopic melanocytes that have undergone malignant transformation [4, 5].

There is no consensus regarding the diagnosis of PHM. Contemporary diagnostic criteria include the following: 1) hepatic melanoma confirmed histologically and immunohistochemically, 2) exclusion of other primary malignant melanomas, and 3) the absence of a previous cutaneous tumour (that was destroyed or excised without histologic examination). It is difficult to identify this disease without histologic or immunohistochemical results because there is no typical clinical picture for patients with PHM. In our experience, certain imaging findings on CT or MRI suggest a PHM: 1) a detectable pseudocapsule around the liver mass; 2) slight enhancement of the mass during both arterial and portal venous stages; and 3) on MRI, the mass tends to present as a heterogeneous hyperintense lesion on T1WI and as a hypointense lesion on T2WI. However, these imaging findings only lead us to a presumptive diagnosis of PHM; histology and immunohistochemical staining remain the 'gold standard' criteria for the definitive diagnosis of PHM.

Both of our cases were preoperatively misdiagnosed as hepatocellular carcinomas. The clinical, laboratory, and imaging findings were so nonspecific that it was difficult for the surgeons to make a diagnosis without pathological results. The accurate diagnosis of these lesions without pathological results remains an ongoing challenge to be solved in the future. Our experience demonstrates that, for liver masses with the imaging findings mentioned above, a diagnosis of PHM should be considered.

The optimal treatment and the prognosis for PHM remain largely unknown. Surgical resection has been well documented to be the best choice for treating isolated hepatic melanoma metastases [6–9]. Based on our results, we hypothesise that surgical intervention may also be the best choice for patients with resectable PHM. We performed R0 resections with lymph node resection in the hepatoduodenal ligament in both of these cases. The tumour number, size, location and treatment were similar in the two patients. However, dramatically different survival times were achieved. The first patient died 4 months after surgery, while the other remains disease-free up through the present (12 months after surgery). Our results suggest that complete surgical extirpation may be potentially curative in some cases (case 2). Another point is that tumour rupture, which is correlated with intraperitoneal metastasis, is an important factor influencing survival in patients with PHM (case 1). In both cases, we found enlarged lymph nodes in the hepatoduodenal ligament, indicating that PHM has a high incidence of lymph node metastasis. We suggest that R0 resection of the hepatic tumour with lymph node resection in the hepatoduodenal ligament is necessary for the radical resection of PHM.

Until now, no conventional treatments have shown efficacy in the treatment of PHM. Large doses of IL-2 have been suggested by the National Comprehensive Cancer Network as the treatment of choice for advanced melanoma. Chemotherapy (dacarbazine) has shown limited efficacy [10]. However, several new molecular targeted therapies such as nivolumab and ipilimumab have been applied in phase III trials with encouraging results [11, 12]. Additionally, some doctors have reported that treatment with surgical resection followed by adoptive cell therapy shows some benefit for patients with malignant melanoma [9].

## Conclusion

We present two cases of PHM, a rare disease that has been addressed by few reports in the past. Primary hepatic melanoma should be considered as a diagnosis in patients with the imaging findings described in this report. For appropriately-selected PHM patients, surgical resection represents a feasible, safe and potentially curative treatment option.

## Consent

Written informed consent was obtained from the patient for publication of this case report and any accompanying images. A copy of the written consent is available for review by the Editor-in-Chief of this journal.

## Abbreviations

AFP: alpha-fetoprotein
CK: cytokeratin
CT: computed tomography
IL-2: interleukin-2
MRI: magnetic resonance imaging
PET-CT: positron emission tomography-CT
PHM: primary hepatic melanoma
T1W1: T1-weighted imaging
T2W1: T2-weighted imaging.
